# Patient Advocacy in Transforming Surgical Lung Cancer Care in Europe

**DOI:** 10.1093/icvts/ivag012

**Published:** 2026-01-08

**Authors:** Cecilia Pompili, Antonio Ungaro, Korina Pateli-Bell, Shani Shilo, Merel Hennink, Yvonne Diaz, Stefania Vallone, Silvia Novello, Debra Montague

**Affiliations:** Institute for Clinical & Applied Health Research, University of Hull, Hull HU6 7RX, United Kingdom; Medical Oncology Unit, San Giuseppe Moscati Hospital, Statte, Taranto, 74010, Italy; FairLife Lung Cancer Care, 18 Napoleontos Zerva Street, Glyfada, 16675, Greece; The Israeli Lung Cancer Foundation, Rehovot, 7639302, Israel; Stichting Merels Wereld, Groningen, 9722 PL, The Netherlands; Oncogene Cancer Research, London, W11 4QX, United Kingdom; Women Against Lung Cancer in Europe Onlus, Orbassano, Turin, 10143, Italy; Department of Oncology, University of Turin, AOU San Luigi Gonzaga, Orbassano, Torino, 10143, Italy; ALK Positive UK, Surrey, NP15 2FD, United Kingdom

**Keywords:** patient advocacy, non-small cell lung cancer (NSCLC), early-stage, thoracic surgery, perioperative management, neoadjuvant/adjuvant therapy, multidisciplinary care, health equity

## Abstract

**Objectives:**

To provide a European-focused overview of the role of patient advocacy groups in shaping surgical lung cancer care, highlighting their contributions to multidisciplinary care, equitable access, psycho-social support, and patient-centred research.

**Methods:**

We conducted a narrative review of the major European and national lung cancer advocacy organizations, integrating perspectives from patient leaders and thoracic surgeons. The analysis focused on initiatives directly impacting thoracic surgery and perioperative care, with attention to education, prevention, survivorship, and research advocacy.

**Results:**

Multiple advocacy organizations have significantly influenced lung cancer care with relevance to surgery. Oncogene Cancer Research (UK) promotes transparent information and shared decision-making around surgical options across all disease stages. Women Against Lung Cancer in Europe delivers large-scale initiatives such as European Program for ROutine testing of Patients with Advanced lung cancer, expanding molecular testing and psycho-social support across several European countries. The Israeli Lung Cancer Foundation secured national low-dose CT screening and mandatory multidisciplinary team review for early-stage patients. In Greece, FairLife launched the BREATH program, providing structured psycho-social support integrated with surgical pathways. Longkanker Nederland advances shared decision-making through national decision aids, patient-reported outcomes, and guideline development. ALK Positive UK develops tailored education for patients and clinicians, addressing the impact of biomarker status on surgical pathways. At the European level, Lung Cancer Europe drives large-scale surveys, awareness campaigns, and collaborations with European Society of Thoracic Surgery to embed patient perspectives into surgical discussions.

**Conclusions:**

Patient advocacy is increasingly shaping thoracic surgery in Europe, bridging gaps in communication, equity, and research. By collaborating with advocacy organizations, surgeons can deliver more integrated, communicative, and patient-centred care, ensuring that surgical innovation aligns with the lived experiences and priorities of patients.

## Graphical abstract

**Figure:**
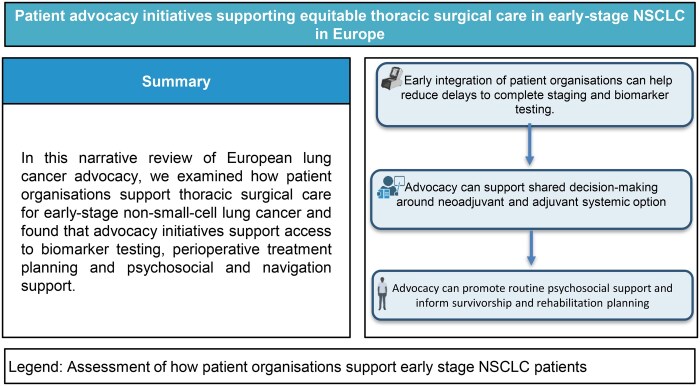

## INTRODUCTION

Patient advocacy in lung cancer has driven transformative changes in clinical practice over the past 3 decades. Patient advocacy is an underutilized tool that thoracic surgeons can use to improve patient care and the profession.

Advocacy should be part of a physician’s knowledge and curriculum, regardless of specialty.

In lung cancer care, early advocacy efforts—such as the Alliance for Lung Cancer Advocacy, Support, and Education founded in 1995, played a pivotal role in destigmatizing the disease and promoting its inclusion in national research agendas.^1^ In the 2000s, groups like LUNGevity and the American Lung Association drove the adoption of low-dose CT (LDCT) screening, increasing early-stage diagnoses and supporting the US Preventive Services Task Force recommendations through educational tools such as Project ACTS.^2^^,^^3^ These initiatives have supported the implementation and uptake of lung cancer screening programs, helping to raise awareness and increase early-stage diagnosis rates, rather than directly influencing population-level mortality reductions.^4^

With the advent of molecular oncology, patient-led groups (eg, ROS1ders, EGFR Resisters) prioritized genomic testing and co-developed research tools, such as global registries tracking biomarker-driven outcomes.^5^^,^^6^ Their advocacy enabled broader access to targeted therapies like Crizotinib and Osimertinib.^7^^,^^8^ Moreover, initiatives such as the International Association for the Study of Lung Cancer (IASLC) Patient Advocate Committee initiated projects on the implementation of patient-centric end-points (eg, quality of life) into clinical trials.^9^

Advocacy has also influenced policy and funding. Campaigns like “Saved by the Scan” improved access to screening for underserved populations, while lobbying increased federal research funding from USD 331 million (2016) to over USD 447 million (2020).^10^ Today, thanks to advocacy, molecular profiling is standard, trial designs are more patient-oriented, and multidisciplinary approaches—especially in perioperative settings—reflect a stronger commitment to shared decision-making.

The purpose of this article is to provide thoracic surgeons with a basic framework of knowledge regarding the importance of patient advocacy and to review recent successes in advocacy related to lung cancer care in Europe.

## METHODS

We conducted a narrative review focused on the role of patient advocacy within thoracic surgical pathways for early-stage NSCLC in Europe. Sources included PubMed/MEDLINE, Embase, Scopus, and the Cochrane Library; guideline/policy sites (ESMO, ASCO, IASLC, WHO/NIH); and reports/websites of major advocacy organizations (eg, Women Against Lung Cancer in Europe [WALCE], Lung Cancer Europe [LuCE], LUNGevity, EGFR Resisters, ALK Positive, ROS1ders). The timeframe spanned January 2010 to June 2025 (English-language focus). Search terms combined concepts for lung cancer/NSCLC with advocacy/patient engagement, surgery/perioperative care, shared decision-making/multidisciplinary team, biomarker testing/NGS, and equity/access. We prioritized peer-reviewed original studies, clinical guidelines/consensus statements, perioperative trial reports (chemo-immunotherapy; adjuvant TKI/IO), and high-quality qualitative/implementation studies; grey literature (advocacy white papers and annual impact reports) was used to describe programs not yet captured in journals. Illustrative case examples were selected for relevance to surgical decision-making, magnitude/transferability of impact, and geographic balance. Where appropriate, evidence strength was annotated using the Oxford Centre for Evidence-Based Medicine levels (2020 update) or the grading reported by the source. Given the narrative design, no meta-analysis or formal risk-of-bias assessment was performed; 2 authors independently screened candidate items and resolved discrepancies by consensus.

Where advocacy is linked to clinical gains, we emphasize implementation and equity outcomes (eg, biomarker completeness, turnaround times, navigation uptake, SDM documentation, PROs) rather than causal oncologic efficacy, unless supported by study-level data.

Accordingly, any potential effects on pathologic or survival outcomes should be considered exploratory. We prioritize implementation and equity measures unless robust trial-level evidence supports a causal interpretation.

### Bridging the gap: the reciprocal impact of patient advocacy and thoracic surgery in NSCLC

**Figure 1.**
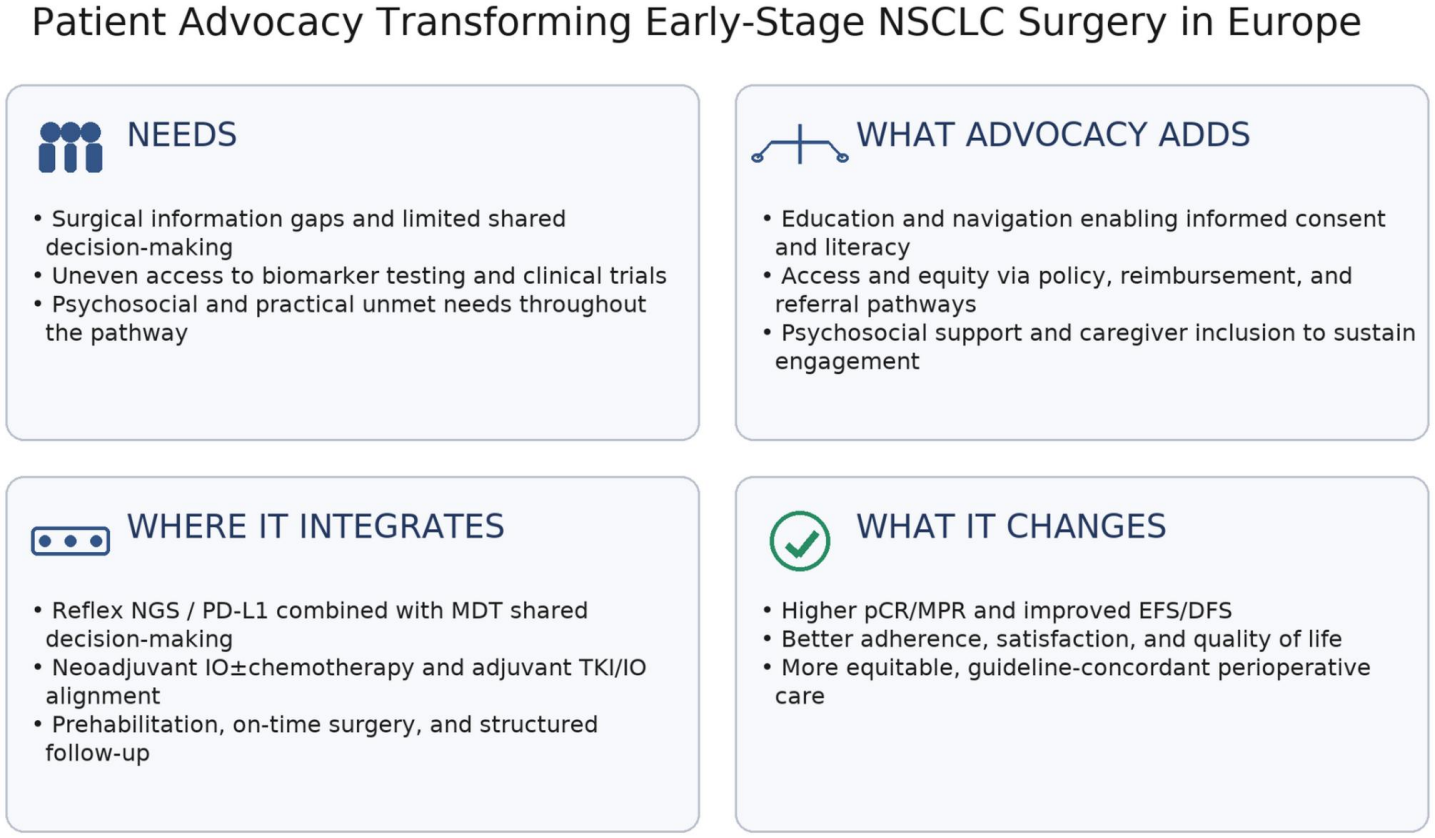
Key Aspects and Role of Patient Advocacy in Early-Stage NSCLC Surgery.

The emergence of multidisciplinary treatment strategies in early-stage non-small cell lung cancer (NSCLC), including perioperative immunotherapy and targeted therapy, has underscored the need for a more integrative approach to care, that meaningfully includes thoracic surgeons alongside medical oncologists, radiation oncologists, pathologists, and, increasingly, patient advocacy groups. Traditionally, advocacy in lung cancer has largely centred on patients with advanced or metastatic disease, where systemic therapy was the mainstay of care and the trajectory of the illness more predictable in terms of advocacy needs. However, as the landscape shifts towards incorporating neoadjuvant and adjuvant therapies within curative-intent treatment pathways, the involvement of thoracic surgeons in patient-centred communication and shared decision-making becomes increasingly critical and so does the role of advocacy in bridging communication and educational gaps between patients and surgical teams^3^ (**Figure 1**).

Advocacy organizations can play a unique role in fostering dialogue between patients and surgeons, helping to demystify the surgical process and support patients in navigating complex treatment choices. In the context of perioperative immunotherapy, patients often face tight treatment timelines and multidisciplinary consultations within a compressed preoperative window.^11^^,^^12^ Advocates can empower patients by providing curated educational materials about the potential risks and benefits of surgery after neoadjuvant immunotherapy, including possible alterations in surgical approach due to immune-related tissue changes, delays in recovery, or risk of incomplete resection.^13^ This level of proactive education not only improves patient understanding and consent quality but also relieves the informational burden that often falls solely on the surgical team during high-pressure consultations.^14^

Conversely, advocacy initiatives can also help sensitize the surgical community to the evolving expectations and psycho-social needs of today’s oncology patients.^15^ The surgeon–patient relationship, once strictly procedural, is increasingly influenced by patient values, long-term quality of life considerations, and survivorship goals, domains traditionally championed by advocacy groups.^16^ By encouraging surgeons to view themselves not merely as technical experts but as integral partners in a longitudinal care continuum, advocacy efforts can foster a shift towards more relational, communicative surgical practice.^17^

This is particularly relevant in the setting of lung cancer, where the psycho-social toll of diagnosis, stigma associated with smoking, and existential anxiety surrounding thoracic surgery are profound.^18^ Advocacy networks offer a platform to address these concerns early and often**—**through peer-to-peer mentorship, support groups, and prehabilitation counselling**—**that can complement the clinical dialogue surgeons are expected to lead.^19^

Although signs of efficacy (eg, pCR/EFS) come from the studies themselves, advocacy plays a role in raising lung cancer awareness and implementation: adherence to the treatment pathway, timely sequencing, quality of informed consent, and equitable access to the indicated regimens.

Moreover, advocacy organizations have the potential to facilitate research collaborations with surgical teams, particularly in areas such as surgical outcomes post-immunotherapy, real-world feasibility of perioperative protocols, and patient-reported experience measures. In doing so, they act as a catalyst for a more inclusive model of translational research, one that places patient priorities and lived experiences at the centre of surgical innovation.^20^ Recognizing that institutional resistance, resource constraints, and variability in surgeon engagement can impede this integration, we recommend a modular, context-dependent framework—MDT charters with defined advocacy roles and a surgical liaison, reflex biomarker testing with patient navigation, and SDM training with audit-and-feedback, tracked by equity-stratified process metrics (biomarker completeness, SDM documentation, prehabilitation uptake).

### No stage left behind: empowering lung cancer patients to discuss surgery—a viewpoint from oncogene cancer research

Patient advocacy has transformed many corners of lung cancer care—expanding access to biomarker testing, targeted therapies and better information. Yet when it comes to thoracic surgery—representing an important chance for a cure—patient voices remain largely absent. Surgery is still one of the least explained parts of cancer treatment.

Oncogene Cancer Research, a UK-based patient and care partner-led charity focused on education, advocacy and support for research, believes this must change. Three gaps urgently need fixing.

#### ALL stages: the lack of clear honest information about surgery

Across all stages, patients lack trusted practical information about surgical options to help them ask the right questions and understand how surgery might fit with other treatment options for their particular oncogene biomarker, their individual stage of disease and their overall health. People with oncogenic drivers are often younger, have few or no other health conditions and deserve tailored conversations about what’s possible and appropriate for them.^21^ No one should face life changing choices without plain language information resources and tools and be without a seat at the table.

#### Early-stage patients: the cured but forgotten

People diagnosed with early-stage lung cancer often describe being “cured but forgotten.” Many hear the shocking words “you have cancer,” endure major surgery, then hear the welcome but abrupt: “You’re cured. Get on with your life.” In the UK and many other countries, these patients rarely see an oncologist as they are treated in the curative setting.^22^ There is little chance to discuss whether a biomarker was found, what it means if the cancer returns, or how to plan for the future. Many chase follow-up scans alone, unsure who to call with concerns. As Angela, a Stage 1 A survivor, puts it: “The journey’s not finished after surgery. It marks the start of years of scans and worry—but no real support.” NICE’s lung cancer guideline (NG122)^23^ rightly covers surgery for early stages, but says little about survivorship, biomarker-informed monitoring or the mental toll of uncertainty.

#### Advanced or inoperable patients: no guidelines, no conversation

For people with more advanced or inoperable lung cancer—from stage IIIb to stage IV—the silence is different but just as damaging. Internationally, new guidance from groups like the Society of Thoracic Surgeons recognizes that for some carefully selected patients, surgery may help as part of a multimodal approach.^24^ But in the UK, NICE offers no clear pathway. With no national guidance, surgery is rarely discussed and quickly dismissed. This leaves some patients to push for options on their own, against deep-rooted assumptions that advanced means inoperable. As Steve, a molecular biologist and paramedic with stage IV ALK+ lung cancer who underwent a 2 year-long, stressful process to undergo surgery put it: “I knew I needed to be my own advocate.” It is an unrealistic burden for patients—one that fuels delays, stress and financial burden, and, in some cases, forces patients to seek private surgery they cannot easily afford.

Oncogene Cancer Research believes patients must be equipped to take part in shared discussions and decision-making about surgery—so that practices evolve, choices are clearer, and no one is left in the dark.^25^

### European context and generalizability

Recommendations anchored in pan-European guidance (eg, biomarker reflex testing, MDT working, patient-centred consent and PROs) are broadly generalizable across EU/EEA settings. Country-specific examples (such as national screening pilots, advocacy-led navigation programmes, and legal support services) illustrate feasible pathways within diverse health systems but still require adaptation to local funding and governance structures.

Importantly, the role of patient organizations must also align with these national and European contexts; this is precisely where LuCE plays a distinctive role, helping to connect and harmonize the efforts of individual organizations across Europe.

**Table 1** maps each advocacy example to its primary lever and the corresponding step(s) of the surgical pathway, indicating whether each recommendation is EU-generalizable or country-specific.

**Table 1.** EU Focus Mapping

Recommendation/example	Policy anchor	Scope	Notes for EU/EEA adaptation
Reflex biomarker testing + navigation	ESMO/IASLC guidance (general)	EU-generalizable	Requires lab SLAs, LIS integration, and navigator role within MDT.
Patient-centred consent/SDM materials	NICE/ESMO consent principles (general)	EU-generalizable	Use co-created materials; track SDM documentation and decisional conflict.
Advocacy-supported legal/transport aid	Local NGO programs	Country-specific exemplar	Depends on regional welfare/NGO funding; target underserved geographies.
Prehabilitation + PROs in peri-IO/TKI	ERAS/ESTS concepts + local pilots	EU-generalizable (implementation varies)	Start with pilot clinics; ePROMs embedded in pre-op assessment.

### Women Against Lung Cancer in Europe

Women Against Lung Cancer in Europe is a European non-profit organization founded in Italy in 2006 by a multidisciplinary team. Initially dedicated to raising awareness about the increasing incidence of lung cancer—particularly among women—and to disseminating evidence-based information, WALCE has since evolved into a comprehensive patient advocacy group addressing the broader needs of all individuals affected by lung cancer. Now composed primarily of patients and family members, the organization operates nationally and across Europe through partnerships with scientific societies, healthcare institutions, and other advocacy networks. Over nearly 2 decades, WALCE has launched numerous community-driven initiatives responding to unmet patient needs. Among them, the flagship European Program for ROutine testing of Patients with Advanced lung cancer (EPROPA) program, initiated in 2021 in Italy, Slovenia, Greece, Romania and, recently in Albania, that provides free NGS (next generation sequencing) testing for advanced NSCLC patients and logistical/financial support for clinical trial participation abroad—particularly impactful in resource-limited settings.

The organization offers a wide range of educational and psycho-social support services, including evidence-based materials, psychoeducational events, and “Be Mutual Days,” a 2-day event for oncogene-addicted patients and caregivers designed to enhance knowledge, address shared concerns, and build community. Other tailored interventions include mindfulness courses, make-up workshops, nutritional counselling, adapted physical activity which has demonstrated measurable improvements in quality of life, and the ComuniCARE initiative for young patients with children. Practical and legal support services are also available, such as free legal counselling and transportation assistance for economically or logistically vulnerable patients.

In prevention, WALCE promotes anti-smoking campaigns tailored by age group and advocates for lung cancer screening, supporting Italy’s national pilot program since 2022. Its advocacy efforts also target policymakers to promote equitable access to early diagnosis, innovative therapies, and high-quality care. Through awareness campaigns, short films, and web series, the organization works to reshape the public perception of lung cancer, combat stigma, and highlight progress in research and patient outcomes. WALCE today represents a leading model of integrated patient advocacy in thoracic oncology, combining prevention, education, support, empowerment, and health equity.

### The Israeli Lung Cancer Foundation

In Israel, the Israeli Lung Cancer Foundation, has played a central role in advancing early detection and comprehensive care for lung cancer patients. One of its most impactful achievements has been the national advocacy work for several years, that led to the inclusion of LDCT screening for high-risk individuals as a pilot and then in the government-funded health basket in 2025. This milestone, achieved through years of public awareness, policy engagement, and collaboration with medical societies, has already led to a measurable rise in early-stage lung cancer diagnoses.

Since the program’s implementation, the foundation has seen a growing number of early-stage patients—particularly those diagnosed at Stage I or II—reach out for support.

To address this, the foundation developed a patient navigation model that provides emotional, logistical, and informational support. Patients are encouraged to meet with an oncologist in addition to a surgeon, to ensure they understand the full range of evidence-based treatment options. This approach has been reinforced at the national level: the Israeli Ministry of Health recently issued a formal guideline requiring that all early-stage lung cancer cases be reviewed in a multidisciplinary team (MDT) meeting. This ensures input from thoracic surgeons, oncologists, pulmonologists, radiologists, and pathologists when treatment plans are being developed.

Another strength of this evolving model is the growing cooperation between the foundation and the thoracic surgical community. Surgeons across Israel are increasingly engaged with the foundation’s advocacy and education work, often referring patients directly to ensure they are informed, supported, and connected to the wider care system.

Together, these efforts are creating a more integrated and patient-centred approach to early-stage lung cancer. The combination of national policy change, proactive patient support, and stronger collaboration with healthcare professionals is setting a new standard of care. The Israeli Lung Cancer Foundation continues to demonstrate that advocacy can meaningfully influence both national policy and individual patient outcomes—especially for those whose cancer is detected early, when the chances of cure are highest.

### The need of psychosocial support during the pre-peri-postoperative period in Greece and the FairLife initiatives

In Greece, the provision of psycho-social support for lung cancer patients, particularly during the preoperative, perioperative, and postoperative periods, is insufficient. While much focus is placed on medical treatment and surgery, emotional and psychological support often remains an afterthought. Many patients report feeling neglected in this area, with little attention given to the mental and emotional toll of the disease and its treatment.

Several patients have shared personal stories that highlight the lack of psychological care. The long wait times for surgery due to lengthy waiting lists in public hospitals worsens this problem. These delays increase the psychological burden in addition to the risk for patients with lung cancer, whose malignancy is frequently aggressive. When patients experience long waits for important medical procedures, the anxiety and uncertainty associated with receiving a cancer diagnosis can increase due to the lack of timely mental health support.

Integrating psycho-social support into the entire surgical process, encompassing preoperative counselling, emotional support during surgery, and postoperative care, could significantly improve patient outcomes. Not only would this ensure that patients feel more prepared and supported, but it could also enhance recovery and long-term well-being.

In response to this unmet need, FairLife Lung Cancer Care developed and offers the BREATH Psychosocial Support Program–a pioneering, nationwide initiative launched in 2022 entirely free of charge. BREATH provides structured psychological support to people affected by lung cancer, including patients, survivors, caregivers, and the bereaved, through individual counselling, group sessions, grief support, and therapeutic activities. The program is designed to be accessible, stigma-free, and personalized, addressing the emotional, mental, and social dimensions of the disease.

To date, BREATH has supported more than 1105 individuals, delivering over 6149 hours of psychological care, both in-person and online. Many participants include surgical patients who struggled with fear of recurrence, post-traumatic stress, or the emotional consequences of physical changes after surgery. The program’s impact is evident in the testimonies of participants who report increased resilience, reduced anxiety, and improved quality of life. BREATH is an example of how psycho-social support, when made a fundamental part of the care journey, can transform patient outcomes and restore dignity and hope in the face of lung cancer.

In collaboration with surgical departments and healthcare professionals, FairLife has helped to design support structures that focus on both medical treatment and psycho-social care. That marks the only way to ensure these efforts provide patients with comprehensive care, addressing not only their physical health but also their emotional needs throughout their treatment.

### The Dutch experience—Longkanker Nederland Organization

In the Netherlands, nationwide standards provide a structured care pathway for patients referred to pulmonologists with suspected lung cancer. Diagnostic tests are scheduled promptly, and pulmonologists coordinate care. Surgical cases are discussed in regional or local multidisciplinary meetings, and patients are informed of the treatment plan, and retain the right to decline at any stage.

There is a very progressive attitude among surgeons, with active collaboration between them and the national patient organization, enabling shared decision-making. The patient organizations are involved in multiple initiatives to improve lung cancer care, including the development of the lung cancer decision aid, now available for all types and stages of non-small cell lung cancer. It also participates in studies such as ERATS and OptriAL.^26^

Following discharge, patients return to their referring pulmonologist for pathology review and follow-up care. The Dutch Lung Cancer Audit—Surgery (DLCA-S) now incorporates patient-reported outcomes, enabling benchmarking for survival, complications, and patient experience.

National efforts include the creation of the guideline “Optimization of Care for Lung Resections” and a dashboard to monitor the implementation of ERATS in clinical practice, to which the surgeons have contributed. Additionally, surgeons initiated a multidisciplinary collaboration to enhance research cooperation within the Netherlands.

Several Dutch hospitals, including Antoni van Leeuwenhoek, Radboudumc, and UMCG, systematically integrate PROs into routine practice, multidisciplinary meetings, and shared decision-making. Patient advocacy continues to play a critical role in ensuring equitable access to specialized thoracic surgery, referral pathways, and postoperative rehabilitation, particularly for patients from underserved regions.

The Health Outcomes Observatory (H2O) project provides standardized infrastructure for patients to measure outcomes while maintaining control of their data, supporting healthcare innovation and improved outcomes across Europe.

### The ALK positive UK experience

ALK Positive UK focuses on raising awareness and improving outcomes for patients affected by ALK-positive lung cancer.

ALK Positive UK is a patient support and advocacy organization covering the UK and Ireland, currently comprising over 780 members, including ALK-positive lung cancer patients and their carers. The organization is recognized by NICE as the voice of ALK-positive lung cancer patients and has contributed to several NICE submissions over the past 6 years.^27^

A key aspect of ALK Positive UK’s advocacy is the development of tailored educational resources for both patients and healthcare professionals. Currently, a significant gap exists in the availability of specialized information regarding how ALK-positive status can influence surgical decisions and post-operative care. Many surgical patients may not receive adequate guidance on the implications of their genetic status, potentially leading to delays in optimal treatment pathways.

To address this, ALK Positive UK sees an opportunity to enhance collaboration with surgical teams to ensure that clinicians are well-informed about the specific challenges and considerations for ALK-positive patients. The organization aims to develop comprehensive guidelines to educate surgeons on personalized treatment approaches, thereby improving patient outcomes and satisfaction.

### Lung cancer Europe perspectives

As a leading European advocacy organization, LuCE continues to build on its core mission while also embracing new directions across Europe and in close collaboration with the other Organizations (**Table 2**). LuCE is shifting from simply highlighting inequalities to actively challenging them through evidence, collaboration, and lived experience.

**Table 2.** European Patient Advocacy Initiatives Mapped to the Surgical NSCLC Pathway—Primary Levers, Targeted Steps, and Implementation Metrics

Organization	Country/scope	Primary lever	Targeted surgical pathway step(s)	Reported outputs/metrics	Evidence type
WALCE/EPROPA	Italy, Slovenia, Greece, Albania, Romania (EU focus)	Education/Biomarker access/navigation (free NGS testing; logistical support for cross-border trial access)	Pre-op decision & MDT planning(biomarker completeness; trial referral)	% NGS completed; biomarker TAT; # cross-border trial referrals; navigation uptake	Program report/observational
LuCE	Pan-European (EU/EEA)	Education/policy/PROs (annual surveys; equity & mental-health focus; advocacy briefs)	Referral & survivorship planning(flag inequities; inform MDT resources and support pathways)	Survey-based access indicators; uptake of recommended supports; PROs awareness	Cross-sectional surveys/policy reports
ALK Positive UK/Europe	UK/Europe	Education/SDM/trial navigation (co-created consent aids; trial signposting)	Consent visit & trial referral (peri-IO/TKI expectations; eligibility awareness)	SDM documentation rate; time to trial screening; patient satisfaction	QI/implementation
EGFR Positive UK	UK (EU collaborations)	Consent & peri-adjuvant literacy(patient-friendly materials; adherence support)	Pre-op consent & adjuvant planning (TKI expectations, toxicity, follow-up)	Decisional conflict score; adjuvant adherence proxies (attendance, refill)	QI/implementation
The ROS1ders (EU cohort)	Global with EU reach	Biomarker literacy/registry (outcomes registry; testing campaigns)	MDT planning (ensure ROS1 testing; integrate results before surgery)	Registry enrolment; biomarker completeness; time to molecular result	Registry/observational

Abbreviations: EU/EEA, European Union/European Economic Area; HRQoL, health-related quality of life; IO, immuno-oncology; MDT, multidisciplinary team; NGS, next-generation sequencing; Pre-op, preoperative; PROs, patient-reported outcomes; QI, quality improvement; SDM, shared decision-making; TAT, turnaround time; TKI, tyrosine kinase inhibitor.

The tenth LuCE survey on mental health in lung cancer—now closed—represents the largest global study to date (*n* = 2204 across 31 countries); findings were launched at ESMO 2025.^28^

Understanding patient needs across the care pathway requires more than assumptions–it demands listening and meaningful response. Now in its fifth year, the Get Checked! campaign has evolved under the theme “Get Supported!,” focusing on life after diagnosis and what is needed to live well with lung cancer.

LuCE also supported a European survey on communication in thoracic surgery and helped bring the patient voice into the conversation through a dedicated session at European Society of Thoracic Surgery (ESTS) 2025. As surgery remains a key part of lung cancer treatment, integrating patient perspectives is essential for advancing truly person-centred care.

## DISCUSSION

The increasing engagement of patient advocacy in surgical decision-making is not occurring in isolation. It is accompanied—and in many cases propelled—by a profound evolution in the attitudes and communication strategies of thoracic surgeons themselves. Historically, the role of the surgeon in oncology was largely confined to the technical execution of resections, often within a narrow communicative framework and with limited involvement in long-term care planning.^29^^,^^30^ However, the complexity of today’s multimodal treatment paradigms demands more than surgical skill, it requires relational competence, empathy, and openness to multidisciplinary collaboration. These qualities are not merely desirable; they are essential to achieving optimal outcomes in a therapeutic era defined by uncertainty, rapid innovation, and rising patient expectations.^31^ This shift in sensibility is increasingly visible in the perioperative setting, where the surgeon is no longer a solitary actor, but part of a complex decision-making network.^32^ In this context, meaningful preoperative communication becomes as critical as technical expertise. Patients facing surgery after systemic therapy may experience heightened anxiety, variable expectations, or fragmented understanding of their therapeutic journey. Surgeons who embrace transparent, empathetic communication are better positioned to build trust and align patient hopes with clinical realities. Patient advocacy groups play a pivotal role in reinforcing this transformation. By equipping patients with evidence-based information, peer support, and advocacy narratives, they elevate the baseline of patient knowledge before the surgical consultation even occurs. This pre-engagement enables more sophisticated and meaningful conversations between patients and surgeons. But just as importantly, it also fosters a virtuous cycle: when surgeons recognize that patients arrive well-informed and eager to participate in decisions, they are more likely to adopt inclusive, dialogic communication styles. Thus, the relationship becomes one of shared investment rather than asymmetrical authority.

The emotional state of a patient can directly affect their recovery process, making it vital to address both the physical and emotional aspects of their care. Psychosocial support may help in reducing anxiety, manage stress, and improve patients’ ability to cope with the challenges they face. It also enables patients to adhere more closely to treatment protocols, which can positively impact outcomes and overall well-being. Crucially, the presence of the medical oncologist as a constant partner throughout the treatment continuum enhances this dynamic. Oncologists often serve as bridges between advocacy organizations and surgical teams, translating patient concerns, setting realistic expectations, and helping synchronize systemic therapy with surgical timing.^33^ When surgeons and oncologists jointly communicate with patients, particularly in tumour boards or multidisciplinary clinics, they model the collaborative ethos that advocacy groups promote. This can foster a sense of psychological safety and coherence in the care experience, counteracting the fear and fragmentation that often accompany cancer treatment.^34^ The broader implications of this shift are substantial. As the surgeon–patient relationship becomes more dialogic, patient satisfaction, adherence to treatment, and emotional well-being are likely to improve. Moreover, a collaborative stance fosters a learning environment for both patients and clinicians: patients learn to articulate their values and preferences more clearly, while surgeons learn to integrate these perspectives without compromising oncologic rigor.^35^ Advocacy, in this model, is not an external force but a cultural catalyst, promoting a shared language of trust, respect, and agency within the therapeutic alliance.

Ultimately, this evolution reflects a more holistic vision of surgical oncology, one in which technical precision is harmonized with emotional intelligence and where the voice of the patient is not only heard but integrated from the outset.^36^ It is in this intersection between scientific progress, clinical excellence, and advocacy-driven empowerment that the future of lung cancer surgery will be shaped.

Health advocacy should be recognized as a new discipline within medicine, emphasizing the need for physicians to take a more active role in patient education and decision-making.

To prepare future surgeons for advocacy roles, educational initiatives like the Advocacy Toolbox are being developed for medical students.^37^ These programs aim to equip aspiring surgeons with the skills needed to address health disparities and advocate effectively for their patients and communities. As healthcare continues to evolve, advocacy is becoming an essential component of surgical practice alongside clinical excellence and research.^38^

To some extent, the transition from cancer patient—or caregiver—to advocate may emerge naturally as a response to the experience of cancer. However, in the surgical oncology setting, transforming this personal journey into meaningful, system-level impact requires structured leadership, strategic coordination, and integration within clinical pathways. Harnessing this instinct through organized advocacy is essential to drive improvements in surgical care delivery, patient experience, and equity across the cancer care continuum.

**Figure 2.**
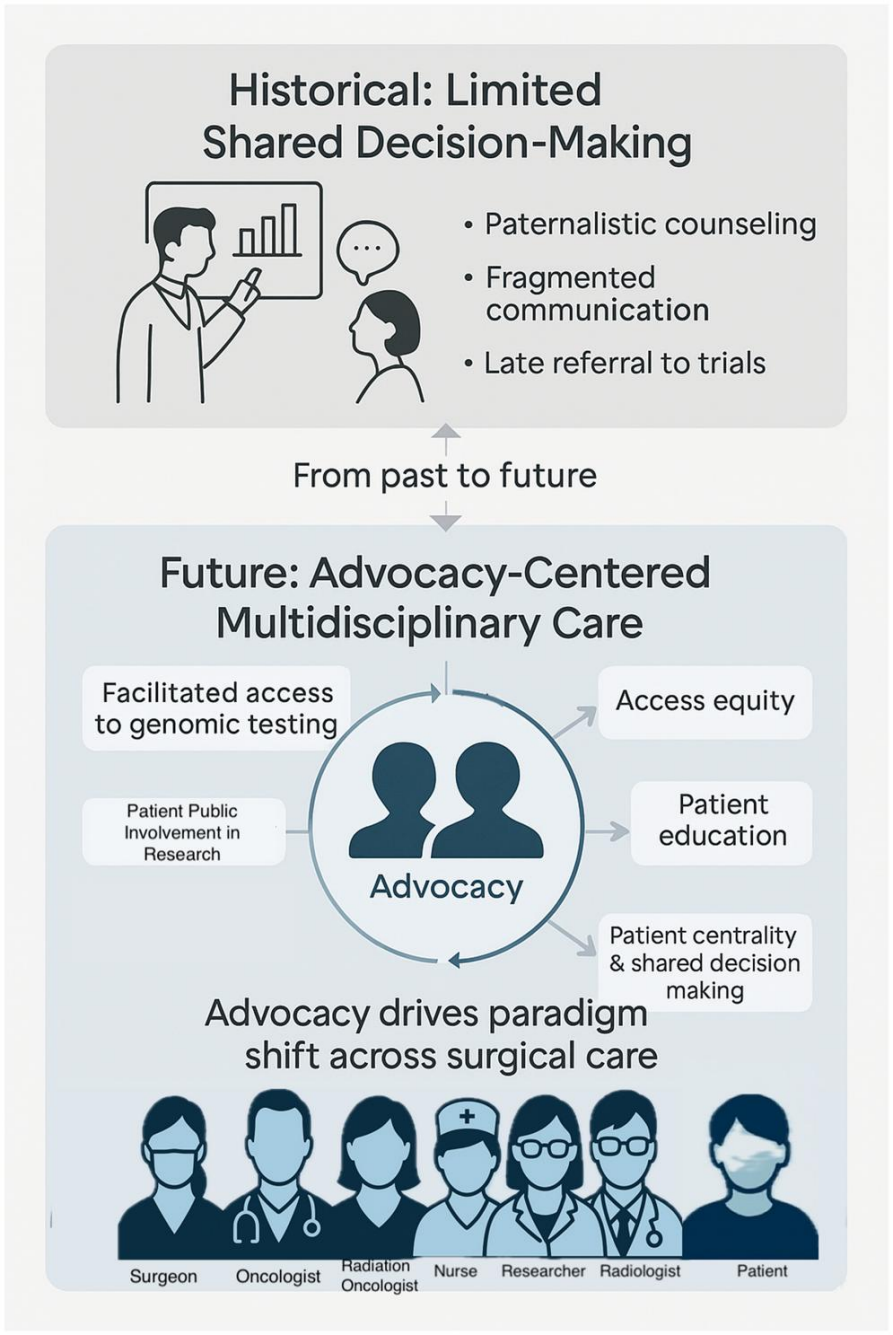
Evolution of Multidisciplinary Care in Early-Stage NSCLC—From Fragmented Pathways to an Advocacy-Centered MDT Integrating Education, Navigation, and SDM. Abbreviations: MDT, multidisciplinary team; NSCLC, non-small cell lung cancer; SDM, shared decision-making.

While advocates are rarely involved in the day-to-day execution of surgical cancer research, their contributions are increasingly recognized in shaping its direction and priorities. Advocates may participate in: (1) guiding the allocation of research funding, (2) contributing as members of multidisciplinary research teams, (3) informing the design and implementation of clinical trials, (4) supporting the translation and dissemination of research findings into clinical practice, and (5) engaging in research governance, policy development and trial recruitment (**Figure 2**).

**Figure 3.**
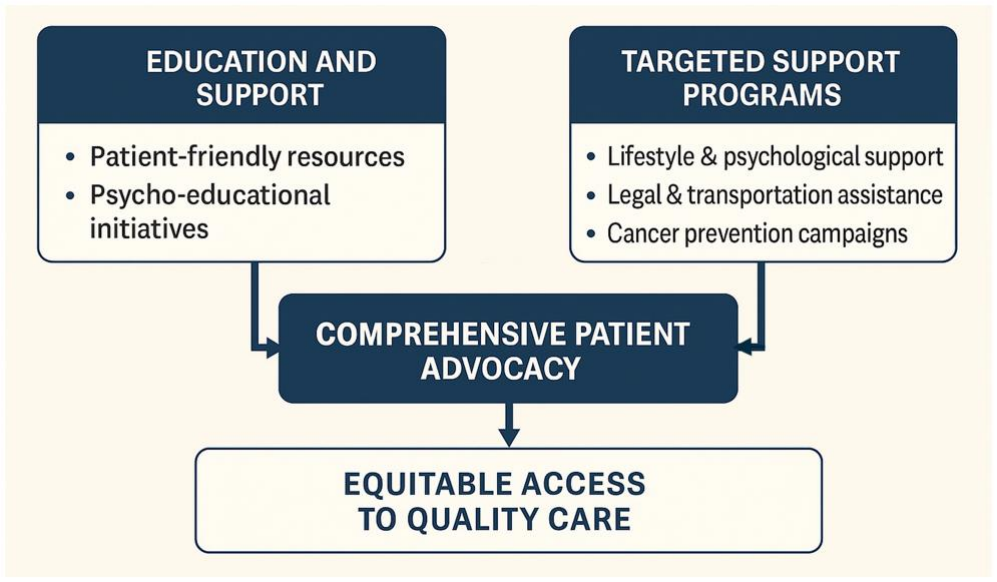
Patient Education/Support and Targeted Programs (Psychosocial, Legal/Transport, Prevention) Converge into Comprehensive Patient Advocacy, Enabling Equitable Access to High-Quality Care.

Patient and Public Involvement (PPI) is increasingly recognized as a fundamental component of high-quality, equitable, and patient-centred research. In the UK, the National Institute for Health and Care Research (NIHR) has developed a clear strategy to embed PPI across all stages of the research process—from setting priorities and reviewing study designs to participating in research teams and disseminating results. The NIHR emphasizes conducting research with and for patients, rather than simply about them, as a way to enhance the ethical foundations, scientific relevance, and real-world impact of health research. Through advisory roles, protocol reviews, trial steering committees, and engagement activities, patients and the public play a vital role in shaping research that better reflects the needs and experiences of those it aims to serve (**Figure 3**).

Many begin their advocacy journey by serving as reviewers of grant applications, particularly for patient-centred or health equity-focused research. This often serves as an entry point, opening pathways to deeper collaboration with surgical research groups and involvement in the development and oversight of clinical trials.

Advocacy plays a crucial role in shaping surgical care by ensuring that patients are not only recipients of treatment but active participants in their care journey. By amplifying the lived experiences of people affected by lung cancer, organizations like LuCE help create a more responsive, equitable, and compassionate surgical environment–one where decisions are guided not only by clinical evidence, but also by what truly matters to patients.

**Table:** 

Advocacy integration for thoracic surgical teams
• Define governance: add advocacy roles to the MDT charter; appoint a surgical liaison. • Practice summary—Advocacy integration for thoracic surgical teams biomarker readiness: reflex testing with lab SLAs; track biomarker completeness/TAT. • Embed navigation: nurse/patient navigation for testing, prehab, logistics, and trial referral. • Standardize consent/SDM: co-create patient-friendly materials; document SDM; monitor decisional conflict. • Equity by design: interpreters, transport vouchers, digital support; equity-stratified metrics(language/region/deprivation). • Measure what matters: prehab uptake, time-to-surgery, cancellations, ePROMs/HRQoL.

## Data Availability

The data underlying this article will be shared on reasonable request to the corresponding author.
